# DIANA, a Process-Oriented Model of Human Auditory Word Recognition

**DOI:** 10.3390/brainsci12050681

**Published:** 2022-05-23

**Authors:** Louis ten Bosch, Lou Boves, Mirjam Ernestus

**Affiliations:** Center for Language Studies, Radboud University, 6525 HT Nijmegen, The Netherlands; lou.boves@ru.nl (L.B.); mirjam.ernestus@ru.nl (M.E.)

**Keywords:** speech comprehension, computational model, process-oriented model

## Abstract

This article presents DIANA, a new, process-oriented model of human auditory word recognition, which takes as its input the acoustic signal and can produce as its output word identifications and lexicality decisions, as well as reaction times. This makes it possible to compare its output with human listeners’ behavior in psycholinguistic experiments. DIANA differs from existing models in that it takes more available neuro-physiological evidence on speech processing into account. For instance, DIANA accounts for the effect of ambiguity in the acoustic signal on reaction times following the Hick–Hyman law and it interprets the acoustic signal in the form of spectro-temporal receptive fields, which are attested in the human superior temporal gyrus, instead of in the form of abstract phonological units. The model consists of three components: activation, decision and execution. The activation and decision components are described in detail, both at the conceptual level (in the running text) and at the computational level (in the Appendices). While the activation component is independent of the listener’s task, the functioning of the decision component depends on this task. The article also describes how DIANA could be improved in the future in order to even better resemble the behavior of human listeners.

## 1. Introduction

This paper presents DIANA, a new, computational model of human speech processing. This model has been developed over a number of years. Implementation details of the model and specific simulations have been described in [1,2,3,4,5,6,7]. The current paper presents DIANA at the conceptual level and explains how its features are inspired by psycholinguistic and neurophysiological data. In addition, it makes explicit how and why DIANA differs from existing models of speech comprehension. Computational details that are relevant for the operation of DIANA are described in the Appendices.

In the following subsections, a number of existing computational models of human speech processing and their characteristics are described. There are more models, such as those based on episodes, but those mentioned here provide a framework for discussion about DIANA’s position. In Section 2, we introduce DIANA and describe how this model differs from existing models at the conceptual level. In Section 3 and Section 4, we describe and illustrate the operation of two of DIANA’s components, while in Section 5 a number of future research directions are discussed.

### 1.1. Computational Models of Speech Processing

A substantial part of psycholinguistic research focuses on the cognitive processes that take place when listeners perceive speech. Based on a vast body of empirical psycholinguistic results obtained since the nineteen-eighties, a number of influential models of human speech comprehension have been developed. These models are based on three basic principles that are assumed to underly human speech processing. These principles are: (1) during the unfolding of the acoustic signal, multiple word candidates are activated in parallel; their activation is based on the degree of match between the input speech signal and their representations in the mental lexicon, (2) this mental lexicon contains information about the pronunciations and meanings of words, (3) the comprehension process is incremental; listeners do not wait until the end of a word before they start interpreting the input.

Most current theories of spoken-word recognition are computationally implemented. Computational models have the advantage that they may be able to simulate the conditions of experiments. They thereby allow a direct comparison between model predictions and behavioral results obtained from human listeners using the same stimuli. An unavoidable potential drawback of any computational model is that various implementational assumptions need to be made that are possibly unsupported by empirical data or are left unspecified by psycho-linguistic theories [8].

#### 1.1.1. Cohort Model

The Cohort model [9,10,11] was one of the first models of spoken word recognition. It used phonemic transcriptions as input and accounted for incremental processing. In this model, spoken-word recognition is modeled as a three-stage process, involving access, selection, and integration. The input is dealt with phone-by-phone. Only words for which the beginnings match with the phonemic transcription of the input speech, aligned from a specific onset, are activated and make up a cohort (access). During processing of the next phone in the input, candidate words that no longer match are removed from this cohort. In the end, only one candidate remains (selection). At that moment, the semantic and syntactic properties of the winning word become available (integration).

A challenge for the Cohort model is that it cannot recover from early local mismatches: for instance, a /k/ instead of /g/ in the input blocks the activation of ’garden’, no matter the support for this word after the /k/. Because the properties of the winning word only become available after selection, the cohort model also cannot use word frequency information during the recognition process. This behaviour is not in agreement with empirical data: many speech comprehension experiments have shown that recovery from errors is possible, and that word frequency has a substantial impact on accuracy and speed (see, e.g., [12] for an overview). Its successor version Cohort II  [13,14] addressed these issues, but a major challenge for the cohort models remained the impossibility of defining activation based on the later parts in the word [15].

The Cohort model, like most models (see below), explains specific aspects of the speech comprehension process at Marr’s computational level [16]. The model assumes that the acoustic signal is converted into a prelexical representation. It is this prelexical representation that is then matched with the words presented in the mental lexicon. In addition, the Cohort model assumes that this prelexical representation consists of phones (or phonemes). The advantage of a prelexical level consisting of categorical units is that the matching of the prelexical representation with the lexical representations is unproblematic. For example, different realizations of /a/ as produced by a male and female speaker, while acoustically very different, can be mapped on the same prelexical unit /a/, which then maps on any lexical /a/. It is unclear, however, how these categorical units are extracted from the acoustic signal because individual sounds are often highly ambiguous. As phone annotation tasks show, listeners can often only solve these ambiguities after they have recognized the word, based on other acoustic properties of the word or based on the linguistic context. The same is suggested by recent neurophysiological studies which indicate that how a phone sequence is recognized is influenced by the patterns in the lexicon from the very start  [17,18,19]. It is therefore not likely that, just on the basis of the acoustic input, categorical decisions on the identity of units are made before lexical access takes place, and it is doubtful whether categorical units are instrumental in the comprehension process proper, e.g., [20].

#### 1.1.2. TRACE

The TRACE model [21] has an entirely different design. It is a connectionist interactive-activation model that consists of three layers: a feature, a phoneme, and a word layer. The input to TRACE consists of a sequence of multidimensional (manually crafted) feature vectors, and each word’s pronunciation in the TRACE lexicon is represented as a phoneme sequence. TRACE activates multiple word candidates that match any part of the speech input in proportion to their degree of fit with the complete input. As a result, partially overlapping words are considered in parallel. After nodes are activated, their activation spreads through the layers (feature nodes spread activation to matching phoneme nodes, phoneme nodes spread to word nodes).

In the TRACE model, inhibition takes place within the phoneme layer and within the word layer; the phoneme with the highest activation suppresses candidate phonemes with lower activations, and idem for words. Finally, the candidate word that matches the input best is ‘recognized’. The activation of a word does not decrease in the presence of mismatching input. In its original version, word frequency was not taken into account, but later versions of TRACE do (see, e.g.,  [22]).

The model includes a ‘lexical feedback loop’, which makes it possible to revise the phonemic interpretation of feature vectors to make these comply with the phonemic representation of words. The use of such a feedback loop was criticized by [23] on the basis of the argument that such a loop would not be necessary and was theoretically unjustifiable. This argument continues to play a role in recent models (see, e.g., [24], and commentaries). Another aspect that received criticism was the implausible architecture of the network—each time the next phoneme in the input is to be processed, the search network has to be entirely duplicated.

#### 1.1.3. Shortlist and Shortlist B

The Shortlist model [23] can be considered a response to the TRACE model. A major aim of Shortlist [23] was to show that the lexical feedback loop in TRACE is unnecessary. Its input consists of a phoneme string (again, handcrafted on the basis of an acoustic signal). It consists of two stages. Shortlist’s first stage consists of an exhaustive serial lexical search, which results in a shortlist of maximally 30 candidate words that match the input processed so far (other candidates are not considered). In the competition stage, these candidate words compete in an interactive-activation network in which the word candidates that receive support from the same sequence of input phonemes are connected via inhibitory links. Mismatches with the acoustic signal do not completely block the recognition of a word but lead to decreasing word activation. The word with the highest activation inhibits candidate words with lower activations, and finally the candidate word that best matches the input is recognized. Shortlist’s interactive activation network is equivalent to the word layer of TRACE. Instead of adapting the existing shortlist, the entire process is repeated with each new phoneme symbol in the input, which necessitates a new shortlist for each input phoneme.

Shortlist B [25] is an updated version of the Shortlist model. The theoretical assumptions underlying Shortlist B are identical to Shortlist, but it implements the word competition as a Bayesian update process. Its input is created as follows: first a phonemic transcription is created (by hand) of the speech signal, after which this transcription is transformed into a sequence of phone–phone confusion probabilities. These phone–phone confusion probabilities (defined over three time slices per phoneme) are derived from a large-scale perception study using gated diphones [26,27]. By using these probabilities as input, instead of categorical descriptions, Shortlist B addresses listeners’ capability to process ambiguous speech signals. Shortlist B incorporates word frequencies as prior probabilities, and deals with matches and mismatches using the framework of likelihoods. There is no inhibition, and there is no feedback in the sense of higher layers modulating computations in lower layers. A drawback of Shortlist B is that it does not specify how it would extract information about phone-phone confusion probabilities from the acoustic signal and instead produces them from combining a phone transcription of the acoustic signal with data from perception experiments. In addition, the strict use of the Bayesian framework leads to a rather particular interpretation of how listeners process novel words: listeners can only process an unknown word after they have produced a prior for the acoustic realisation of that new word.

#### 1.1.4. Fine-Tracker

The Fine-Tracker model [28,29] is based on the principles underlying Shortlist B. This model is specifically developed to account for the role of fine phonetic detail in speech comprehension. It is one of the first models that takes acoustic speech signals as input, rather than some kind of segment-level symbolic transcription. Fine-Tracker is a two-stage model. The first stage uses an artificial neural network (ANN) to convert the acoustic signal into a sequence of articulatory-phonetic feature vectors. In Fine-Tracker’s lexicon, words are represented as sequences of such feature vectors, instead of phone labels. In the lexical representations the phonetic features have values 0 (absent) or 1 (present), or NA (not applicable, for example for the component plosive in a lexical feature vector representing a vowel). Phonetically longer segments are lexically represented by duplication of the vectors of those segments. For instance, the first syllable of the English words ’ham’ and ’hamster’ differ from each other in their lexical representations in that the vowel æ of ’ham’, which is reportedly longer than that of “hamster” [30], is duplicated. The bottom-up ANN outputs real-valued feature vectors for which each component can take any value between 0 and 1. The use of the ANN vectors and the lexicon’s vectors allows feature values to ‘spread’ into neighboring feature vectors through assimilation and co-articulation. Fine-Tracker’s word recognition stage uses a probabilistic word search based on classical dynamic programming to find the most likely word sequence.

Fine-Tracker has the advantage of using a flexible signal representation in the form of feature vectors. TRACE also uses feature vectors, but these are essentially recoded phonemic symbols. Another advantage is Fine-Tracker’s ability to use real speech as input. The model has two disadvantages. The performance of Fine-Tracker crucially depends on the ANN: If the ANN makes an error, Fine-Tracker cannot recover. Finally, the exact definition of the match between full-dimensional estimated feature vectors (by the ANN) and the (possibly partially defined) canonical lexical feature vectors is an unsolved issue, since it is unclear how to faithfully compare distances between fully specified vectors and distances between partially specified vectors in the definition of the match between the input signal and lexical representation.

#### 1.1.5. EARSHOT and LDL-AURIS

Recently, computational models have been proposed that avoid pre-lexical levels consisting of explicit abstract units or phonetic/articulatory features. EARSHOT [24] and LDL-AURIS [31] do so by mapping the acoustic signal directly to vectors in a distributed semantic vector space, instead of to words, as in ‘localist’ models, by using neural networks: a two-layer long short-term memory (LSTM) neural network [32] (which models non-linear mappings) in EARSHOT, and a linear discriminative learner (with a linear mapping) in LDL-AURIS. These models are end-to-end in the sense that they circumvent explicit pre-lexical and lexical representations during the processing of the input; instead, these representations may be implicitly present in the layers of these networks. The semantic target vectors can be defined in different ways, e.g., chosen randomly or based on the outcome of a word-to-vector algorithm (e.g., word2vec [33]).

EARSHOT and LDL-AURIS do not claim to explain all putative cognitive processes involved in speech comprehension. Instead, they aim to serve as a cognitive model of human speech recognition without explicit phonetic training and by replacing words by distributed semantic representations, thereby leaving a word’s articulation entirely unspecified.

## 2. Towards DIANA, A Novel Process-Oriented Model

In the past, the absence of empirical evidence about processes in the brain involved in speech comprehension was a valid argument for limiting models to the computational level. The rapid advancement of brain imaging techniques, and especially the availability of a growing corpus of knowledge derived from electrocortocography (ECoG) recordings, e.g., [34,35], make it possible to develop models that are also realistic at the neurophysiological level. DIANA takes into account the limitations that the ‘wetware’ of the human brain imposes on the type of computational processes than can be implemented [36,37,38]. In addition, it is based on psycho-linguistically motivated principles underlying the group of ‘localist’ computational models (including the Cohort model, Shortlist, Shortlist B and Fine-Tracker). From these ‘localist’ models, DIANA adopts the use of a lexicon, the concept of word activations and the unfolding of word hypotheses in parallel (i.e., the activation of words and competition among words as a function of time). DIANA does not assume a prelexical layer in which hard decisions have to be made about abstract prelexical units before lexical access. Instead, the acoustic signal is converted into representations that are neurophysiologically attested. These representations have a statistical relation with the representations in the mental lexicon.

In contrast to nearly all other models, DIANA is process-oriented by including activation and decision processes about word candidates in line with what we know about the neurophysiological basis of perception (via spectro-temporal receptive fields) and human decision making (ambiguity resolution). This will be elaborated upon in Section 3 and Section 4. DIANA’s behavioral adequacy can be tested as it takes as its input the acoustic signal and produces as its output decisions (e.g., on the identity of a word or on whether the word is a real word) and reaction times. It can therefore simulate a literate adult listener who takes part in a psycholinguistic experiment.

**Figure 1:**
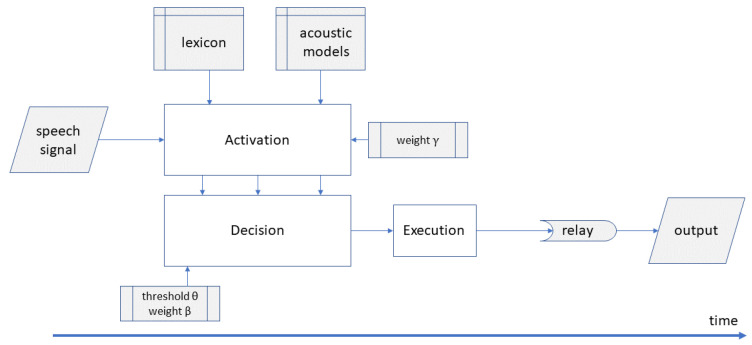
Overall architecture of the DIANA model. The acoustic signal is input for the activation component. During the unfolding of the input, the activation component computes activations and hypotheses which are input for the decision component. The output of DIANA is an overt decision (e.g., word identification or word evaluation) and corresponding reaction time. The two activation and decision components operate in parallel, while the decision and execution components operate serially.

Figure 1 shows the architecture of DIANA. The model contains three interrelated components: an activation component, a decision component and an execution component. The activation component implements acoustic processing and activation of words; the decision component implements the word competition and the decision about the winning hypothesis. The activation and the decision components operate in parallel: the decision component receives a full set of activation scores at each time step from stimulus onset to stimulus offset. The execution component simulates the externalization of the decision, mimicking the time it takes for traveling neural signals to be effectuated eventually as an overt decision. This component adds a constant time (in the current implementation: 200 ms) to DIANA’s RT prediction, and we will not discuss this component further in this article.

## 3. The Activation Component

Given the input speech signal, the activation component computes activations of words in the lexicon, on the basis of which the decision component decides how the input is evaluated (e.g., the identity of the word is established). Before word activations can be computed, the acoustic signal has to be interpreted and represented in such a way that it can connect with the mental lexicon. This section first describes this process, then the assumptions about the mental lexicon, and finally the details of the activation process via a number of examples.

### 3.1. From the Input Signal to Spectro-Temporal Receptive Fields

Experiments producing electrocorticography data (ECoGs, e.g., [34]) with speech input suggest that the neural responses in the primary auditory cortex can be described in the form of so called spectro-temporal receptive fields (STRFs, [39]). STRFs describe the spectro-temporal processing in the human superior temporal gyrus (STG) during natural speech processing (see, e.g., [34,40,41]), and form a neural representation for time-varying sounds, reminiscent of conventional sonagrams [42]. One STRF contains information from both the static spectral (stable portions) and the dynamic spectro-temporal properties (transients) of a short stretch (approximately 20–30 ms) of the speech signal. STRFs also obey the ‘tonotopic’ frequency-locus relation, known from cochlear processing [43].

Approximations of the ‘cortical’ STRFs can be computed directly from the audio signal (see, e.g., [40]). This property is used in DIANA to map the input speech signal into a computational approximation of an STRF sequence in two steps. The first step is the mapping of the input speech to a sequence of feature vectors. Each feature vector represents the static and dynamic part of a 25 ms short stretch of the audio signal. This choice is based on knowledge about temporal alternation of stable regions and transients in speech [44,45,46]. The stable part is coded by 13 Mel-frequency cepstral coefficients, MFCC, [47]. These coefficients take into account the tonotopic properties of cochlear representations and the frequency and loudness sensitivity of the human auditory system (see, e.g., [48]). The dynamic changes of the spectrum are coded by the first and second time derivatives of the MFCCs, cf. [48]. The feature vectors (of dimension 39) are updated every 10ms. Taken together, each audio input is represented by a trajectory of (39-dimensional) feature vectors in the MFCC space, with a sampling rate of 100 per second. Such a trajectory captures the acoustic fine structure of the audio input to a degree that is sufficient for nearly all types of speech analyses [49].

**Figure 2:**
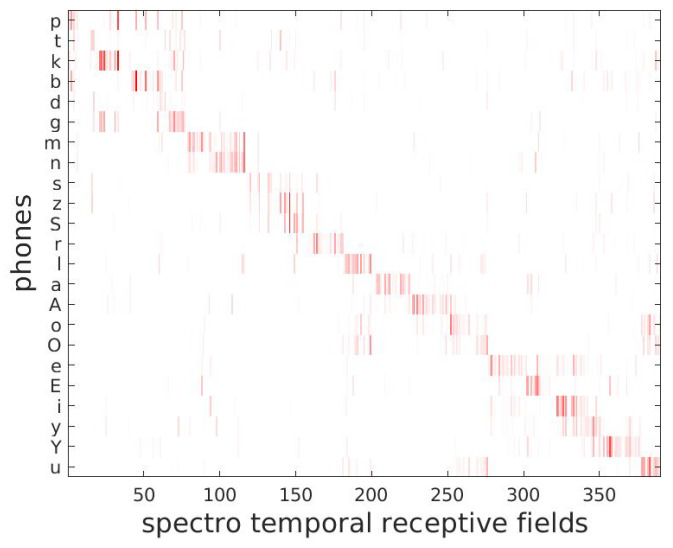
Correspondence between DIANA’s STRFs and frequent phones, organized along broad phonetic classes. Phones are represented using SAMPA.

The second step converts the MFCC feature vectors into the STRFs as used in DIANA. These ‘audio-based’ STRFs are very similar to STRFs based on ECogG data (e.g., [34], see also [50]), and they distinguish phones and broad phonetic classes as the cortical STRFs do (see Appendix A.1 for more details on how STRFs are computed). Figure 2 shows the relation between frequent phones (vertical axis) and DIANA’s STRFs (indexed along the horizontal axis). The off-diagonal cells indicate patterns that are shared among related phones. Importantly, they are very similar to the relation between ECoGs and phones found in neurophysiological studies [34].

STRFs form the link between the pronunciation representations in the lexicon, on the one hand, and the MFCC feature vectors that encode acoustic signals on the other. The match between audio input and a word is computed via the statistical match between the MFCC vectors from the audio input and the STRFs associated with the lexical representation of that word (see Appendix A.1).

### 3.2. The Lexicon in DIANA

DIANA uses an internal lexicon, in which words with their pronunciations are stored. The pronunciations are described in the form of phone sequences. A phone-based representation helps to explain how listeners may divide a word in speech sounds, and it enables DIANA to differentiate between word candidates during the word competition on a phonetically-linguistically relevant level. Another advantage of lexical phone sequences relates to sufficiency; listeners are usually not aware of subtle phonetic differences between different instances of a phone that may arise during speech production.

The lexical representation of words determines how they are modeled in DIANA’s computations. When any two words share a phone with the same pre- and post-context, that phone is modeled by the same articulatory model. For example, since the words ‘speech’ and ‘speed’ share the same word-initial /s p/ in their lexical description, their pre-context is the same (word start) and the post-context is the same (/i/), they share the same /s p/ model. In contrast, ‘spell’, ‘speed’ and ‘speech’ only share the /s/ model, but not the /p/ model,  because the post-context of the /p/ is different in ‘spell’. In the same vein, the words ‘ham’ and ‘hamster’ share the same /h æ/ model, but not the /h æ m/ model. If word stress is not expressed in DIANA’s lexicon, it is not taken into account. That is, words such as ‘household’ and ‘leasehold’ (with stress on the first syllable) share their word-final three-phone model with words such as ‘withhold’, ‘behold’ and ‘uphold’ (with have stress on the second syllable), because the phone representation for the final syllable is the same. Due to the context-dependency, DIANA can process coarticulation effects within a limited scope.

For each (context dependent) phone in the lexical representation, the corresponding articulatory model is a three-state Markov model, in which each state is associated with an STRF. Via self-loop probabilities, the Markov model can deal with duration variation in the input, while the use of three states reflects the head-body-tail structure of the acoustic-phonetic realisation of that unit.

Two observations must be made. First, even though words may share parts of their lexical representations, they can still be in competition with each other. This will be clear from the examples in Section 3.4. Second, the fact that the pronunciation of a word is represented by a sequence of symbols does not imply that these symbols must be (completely) present in the audio input. This flexibility is based on the probabilistic relation between feature vectors (MFCCs) and lexical representations (STRFs) (see Appendix A.1).

### 3.3. Obtaining Activation Scores from Bottom-Up and Top-Down Information

Neurophysiological research using the phonetic mismatch negativity (a measure of mismatch between expected and actual phonetic input) in EEG traces has shown that, from word onset onwards, listeners develop expectations about which word is uttered, based on both the bottom-up information from the acoustic signal, and the top-down expectations from the (linguistic) context [51,52]. In DIANA, the words’ activations are also based on a combination of both types of evidence. The bottom-up support for a word is formed by the match between the MFCC vectors from the audio input with the STRFs associated to the lexical representation of that word (see Section 3.1, and Appendix A.2 for details). The top-down support for a word depends on the task. When a word has to be recognized out of context (e.g., in a psycholinguistic experiment), the bottom-up supports boils down to the word’s frequency of occurrence. In a meaningful context, instead, the top-down information is approximated by the probability of the word given the preceding words, which is computed with a statistical language model (in terms of, e.g., conventional word N-grams).

Since DIANA is a model for spoken word comprehension with as input the speech signal unfolding over time, the activation component does not only assign activations to complete words, but also to cohorts of those words. Longer word candidates match a longer stretch of the acoustic input than short word candidates and, therefore, longer word candidates receive more bottom support. Nevertheless, the input stretch of speech may consist of a series of short words rather than of a long one. In order to compare activations of word candidates with different durations, word activations are normalized by dividing by the word candidate’s duration.

Activations can be computed for words, pseudo-words and parts of words via essentially the same combination of bottom-up and top-down support. Pseudo-words do not appear in the lexicon but obey the phonotactic patterns in the lexicon. They can be neologisms the listener has not heard before, or they can form the pseudo-words in a lexical decision experiment. During the search, DIANA can create pseudo-words as hypotheses on the fly, on the basis of a phone network in which phones are represented as nodes such that only those phone combinations that are phonotactically licensed appear as possible paths through the network. The top-down support for pseudo-words may be very low (e.g., for neologisms in a conversation), but in simulations of experimental outcomes they can be adjusted, e.g., to model the listener’s updated estimation of the proportion of pseudo-words in a lexical decision experiment. Details about the involved computations can be found in Appendix A.3.

### 3.4. Examples of Word Activations

**Figure 3:**
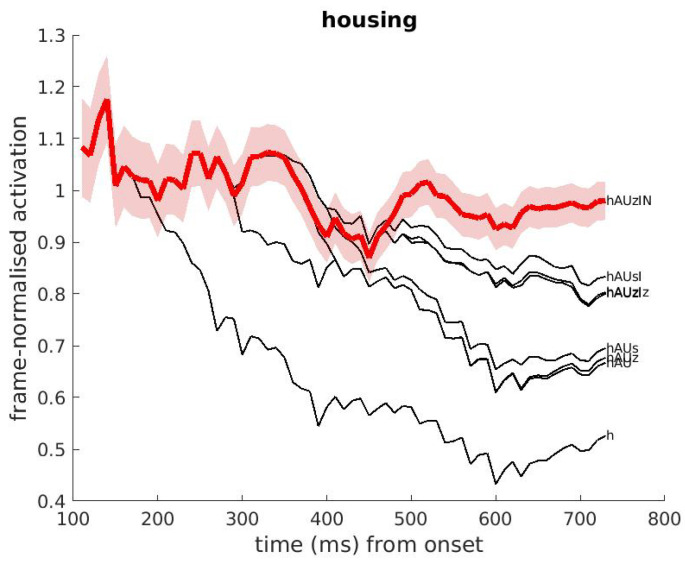
Example of DIANA’s activation over time corresponding to the input English word ‘housing’. The figure shows the activations of the competing words ‘housing’ and ‘houses’, and their word starts (cohorts). The red line shows the evolution of the winning candidate over time. The pink band around the red line indicates the p=0.05 confidence interval. The competing forms are denoted (using SAMPA) at the right-hand side of each plot. A few competitors almost overlap with each other until stimulus offset.

This section presents a number of concrete examples of activations, with emphasis on their evolution during the unfolding of the input signal. The first example, shown in Figure 3, shows the activations of the words ‘housing’ and ‘houses’ and parts thereof, while the speech input is ‘housing’.

In the figure, the vertical and horizontal axes show the frame-normalised word activation and time, respectively. The black traces show the activation of individual cohorts, the phonetic transcription of which (using SAMPA symbols [53]) are shown at the right hand side of the figure. For the sake of clarity, the figure only shows the activations of the words ‘housing’ and ‘houses’ and their cohorts (instead of all words in DIANA’s lexicon). The activation of the word ‘housing’, shown by the red trace, starts to ‘win’ over all other hypotheses at about 500 ms after stimulus onset, and it remains on top until the end of the input. Note that hypotheses that have activations at stimulus offset do not necessarily correspond to existing words, since partial word forms that are part of longer existing words may still be activated on the basis of the complete input signal.

**Figure 4:**
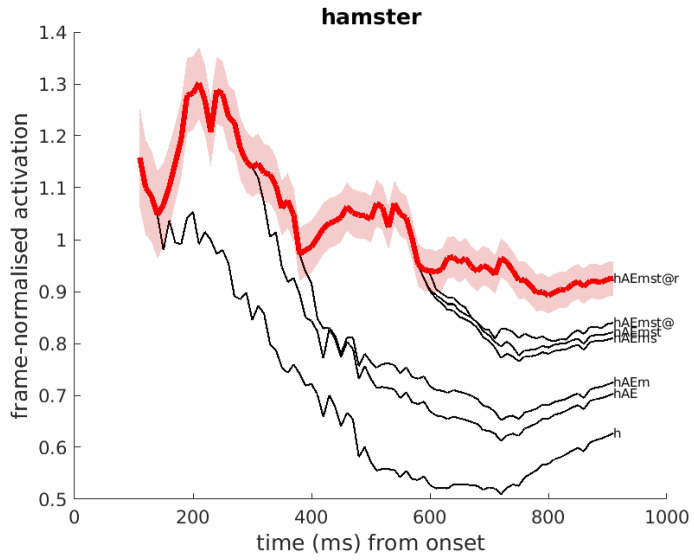
Example of DIANA’s activation over time corresponding to the input English word ‘hamster’. The figure shows the activations of the competing word forms ‘ham’ and ‘hamster’ and their cohorts. The competing forms are denoted (using SAMPA) at the right-hand side. A few competitors overlap with each other until stimulus offset.

Another example is presented in Figure 4, in which the audio input is the word ‘hamster’. At t=380 ms after onset, the competing word ‘ham’ branches of from the winning hypothesis, indicating that the acoustic information disfavours ‘ham’ in the competition with ‘hams’ and other longer cohorts of ‘hamster’. The figure also shows the effect of shared representations of ‘ham’ and the first syllable of ‘hamster’; both activation plots overlap, until t=380 ms.

**Figure 5:**
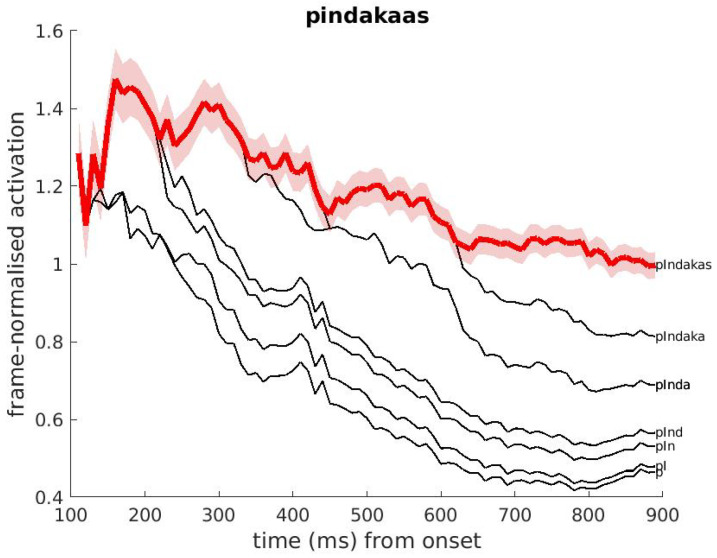
Example of DIANA’s activation over time corresponding to the Dutch word ‘pindakaas’ (SAMPA /pIndakas/; Eng. ’*peanut butter*’). The pink band around the red line indicates the p=0.05 confidence interval.

The following example is in Dutch. Figure 5 presents the activations of the Dutch noun-noun compound ’pindakaas’ (SAMPA /pIndakas/, Eng. ’*peanut butter*’). Certain cohorts of this word are real words themselves, such as the Dutch semantically unrelated word ’pin’ (/pIn/), which can be a noun and a verb form (as its English equivalent ’pin’), and the first constituent of the compound ’pinda’ (/pInda/, Eng. ’*peanut*’). The figure shows that the full word ‘pindakaas’ receives its activation from its cohort /pIn/ until about *t* = 250 ms, while later in the signal, ’pindakaas’ receives it activation from /pInda/. In general, each full form adopts its activation from its shorter cohorts underway, representing the idea that these shorter cohorts are considered as part of the full form under development.

### 3.5. Presence of Noise in the Input

**Figure 6:**
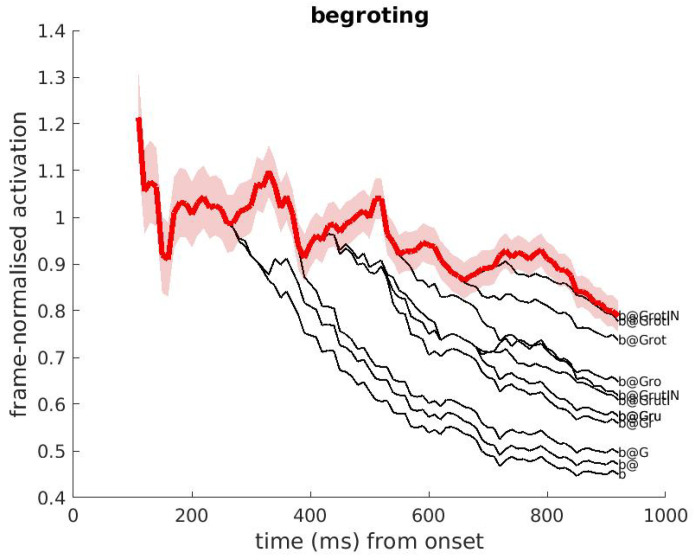
Activation plot of two competing words which form a minimal pair (clean recording condition): the Dutch real word ‘begroting’ (SAMPA /bǝGrotIN/, IPA /bǝXrotIN/, Eng. ‘*budget*’) with ‘begroeting’ (SAMPA /bǝGrutIN/, IPA /bǝXrutIN/, Eng. ‘*greeting*’). The audio is the real word ‘begroting’. The competitor word ‘begroeting’ looses directly after the /o/, at time 450 ms from onset. Clearly, many competitors overlap until the stimulus offset.

**Figure 7:**
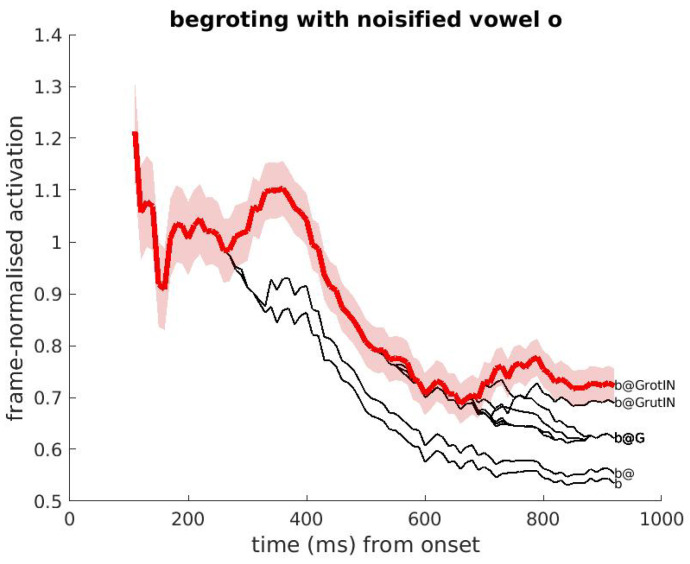
Same as Figure 6, but the noise on the vowel /o/ in the second syllable now yields a reduced and delayed differentiation in the activations of competitors, manifest from 450ms after onset. Many competitors overlap with each other until stimulus offset.

DIANA behaves like humans in that it can recognize words that are partly produced in noise. This can be seen by comparing Figure 6 and Figure 7. Figure 6 (clean condition) shows the competition between the Dutch derived words begroting (/bǝxrotIN/, Eng. ’*budget*’) and begroeting (/bǝxrUtIN/, Eng. ’*greeting*’), which only differ in the vowel in the syllable that carries word stress. As soon as this vowel is processed, the hypotheses bǝxro, and bǝxru, and their longer counterparts, are clearly distinct from each other, showing that activations can differentiate hypotheses on the basis of their final segment.

Figure 7 (noisy condition) shows the activation as a function of time when the word begroting is distorted by superimposing background noise (white noise) on the stressed vowel /o/ in the second syllable with a signal-to-noise ratio of −5 dB. Comparison with the clean condition in Figure 6 shows that the activations are identical between stimulus onset and the noise onset, while soon after the noise onset differences emerge. Compared to the clean condition, the distortion has two substantial effects: first and foremost, the activation score of the ‘correct’ word in the noisy condition shows a steep drop that is completely absent in the clean condition. Second, the divergence between competing cohorts is much smaller in size and occurs later in the noisy condition, compared to the clean condition. (The smaller difference in activation in the case of noise slows down DIANA’s decision, as will become clear in Section 4). In the end, the word is still recognized correctly.

These effects on activation scoress are observed in all cases of segment noisification. Quantitative effects appear substantially stronger in case of distortion of those segments that differentiate between real words, such as the /o/ in begroting versus begroeting.

## 4. The Decision Component

As mentioned above, while the activation component is independent of the listener’s task, the decision component is not. In word identification tasks, it assigns the winning word candidate, while in lexical decision tasks, it determines whether the acoustic input forms a real word or a pseudo-word. In these processes, DIANA only takes into account a selection of the activated words and pseudo-words, as described in Section 4.1. Section 4.2 and Section 4.3 describe the selection procedures in a simple identification task and in a lexical decision task, respectively. In Section 4.4, we discuss situations in which the activation component does not provide sufficient evidence to take a decision.

### 4.1. Selecting Promising Word Candidates

Theoretically, for the purpose of word recognition, the number of potential word candidates can be very large, up to 100,000 words or more. This would correspond to the situation in which a participant is presented a randomly chosen real word. From a neurophysiological perspective, however, it is unlikely that so many competing hypotheses are entertained. For this reason, DIANA reduces the number of activated word and pseudo-word candidates; at each time point, hypotheses with activations too far away (determined by a threshold) from the hypothesis with the highest activation are discarded for further consideration. These hypotheses are considered too poor to have a chance to win later.

As mentioned above, DIANA not only assigns activations to complete words but also to parts of words. This implies that, simultaneously, words and their parts, or parts and their parts, may be activated. For instance, the partial input /k ǝ θ i/ (from “cathedral”) may activate hypotheses such as /k ǝ θ/, /k ǝ θ i/ and /ǝ θ i/. In DIANA, such ‘nested’ candidates (one candidate is a part of the other) are not assumed to be competitors of each other, as they lead to the recognition of the same longer candidate. The decision component therefore ignores all candidates that are part of other candidates with higher activations.

It is worthwhile to observe that this issue is not or cannot be accounted for in the computational models that use a purely symbolic description of the input signal, in which the list of competitors is based on character string comparisons. Neither is it addressed by EARSHOT and LDL-AURIS, because these models do not have a level were such nesting could occur.

### 4.2. The Decision Strategy for Simple Word Identification

The activation data as presented in the Figure 3, Figure 4, Figure 5 and Figure 6 show that the difference in activation between the ‘correct’ word and its best competitor tends to increase (in a non-linear fashion) as the acoustic signal unfolds. Since the activation and the decision components operate in parallel (the decision component receives activations at each time step *t*), the latter component does not have to wait until the end of the word to make a decision.

The decision component selects the word hypothesis with the highest activation, once this activation differs from the activation from the second best word by a certain amount (a threshold θ). DIANA’s use of a decision criterion based on the difference between two activations is commonly used in general models of human decision and RT distributions, for instance, the ballistic accumulation model (BAM) [54] and the linear approach to threshold with ergodic rate models (LATER, [55,56]).

For several reasons, among others between-speaker pronunciation variation and the probabilistic relation between MFCCs and STRFs, it is not guaranteed that the activation for the correct word is always higher than the activation for other words. Inevitably, this may result in word identification errors.

We showed in  [2] that an older version of DIANA can predict identification times for words presented in isolation well. In normal running speech, the role of contextual evidence will be higher than for words presented in isolation, and a substantial difference in activation between the correct word and the competing candidate words is likely to be reached earlier than for words presented in isolation. Context may also inhibit the activation of a candidate word, for example, if the word is unexpected given the pre-context, such that a decision is likely be delayed. Via the Bayes’ formula the pre-context modulates the exact activations of words unfolding over time, and thereby the moment at which the decision component can decide about potential winning hypotheses. Words that receive bottom-up support in line with top-down expectations are responded to more quickly, while words that receive bottom-up information that conflicts with the top-down expectation are decided upon later (to what extent this takes place depends on the effect of this context-modulation on all competing hypotheses).

Many experiments have shown that there is a speed-accuracy trade off; for example, when participants are faster, they tend to be less accurate, and vice versa. In the literature on decision making (see, e.g., [54,57,58,59,60,61,62]), this interaction between speed and accuracy is underpinned by neurophysiological and modeling accounts. In DIANA, the speed-accuracy trade off is the result from a parameter (θ) that determines the value of the threshold difference needed between the activations of the best and the second best word for the best word candidate to be selected. Higher values of θ decrease the risk of making a wrong decision, because more evidence has to be gathered before a decision can be made, which implies longer reaction times. Lower values of θ, instead, increase the risk of making a wrong decision, because less evidence has to be gathered before a decision can be made, which implies short reaction times. The exact speed-accuracy relation depends on the nature (e.g., difficulty) of the task. In [2], we discussed how the threshold θ can affect the speed-accuracy trade off.

### 4.3. The Decision Strategy for Lexical Decision

Participants in a lexical decision experiment may make their lexicality decision, comparing the evidence for the pertinent word to be a real word and the evidence for it to be a pseudo-word. Accordingly, DIANA bases lexicality judgments on the difference in activation between the real word and the pseudo-word with the highest activations. Once this difference has reached a threshold, $\theta_{ld}$, the decision can be made. If the real word has the highest activation, the lexical judgment will be ‘real word’, otherwise it will be ‘pseudo-word’. This decision strategy implies that it is not strictly necessary to decide exactly which word was uttered, but just whether the real word candidate has a higher or lower activation than the pseudo-word candidate.

For a real word as input, DIANA’s competition may involve all lexical items that are acoustically close to the input, in combination with pseudo-words that differ from the input in terms of one or more segments. For example, for an input such as ‘elephant’ (SAMPA: Elǝfǝnt; IPA: εlǝfǝnt), the number of potential competing pseudo-words may easily reach 100 to 200, which is hard to elucidate in a clear picture. Conceptually, it will be clear that the more acoustic information becomes available, the number of viable lexical candidates that are active in the competition will decrease over time. Simultaneously, the number of potential pseudo-words that may play a role in the competition increases over time, due to the increasing length of the hypotheses.

In a lexical decision experiment, the exact nature of the pseudo-words will influence whether participants will make the lexicality decision as soon as the difference in activation exceeds the threshold. If the experiment contains many stimuli that start as real words but turn into pseudo-words only at their final segments, participants may not do so. Instead, they may adopt the strategy to postpone their decisions until they have heard the complete words [63].

Note that a given real word can receive activation as if it is a real word and as if it is a pseudo-word (i.e., via the non-lexically-constrained activations). Importantly, the top-down activation may make the difference; the activation of pseudo-words is only differentiated by the bottom-up activation (since their top-down activation is stimulus independent) while the activations of real words are modulated by top-down information (e.g., the frequency of occurrence of that word). The precise balance between bottom-up and top-down probabilities depends on the listener’s task. In the simulation of a lexical decision experiment during which the listener is confronted with a fifty-fifty proportion of real words and pseudo-words, the priors for ‘word’ and ‘pseudo-word’ will be 0.5. With this decision strategy, DIANA thus is also able to explain a lexical bias that is often observed in psycholinguistic experiments. In [2,3], we analyzed accuracy scores and reaction times from large Dutch and north-American-English datasets of lexical decisions. We showed that DIANA’s decision strategy can distinguish between real words and pseudo-words well and can predict well the lexical decision times (in terms of the Pearson correlation with participants’ reaction times).

### 4.4. Ambiguity during DIANA’s Search Process

As explained above, DIANA can make a decision about the identity of a word or about the lexicality of a stimulus once the difference in activation between two candidates exceeds a certain threshold. In some situations, however, this threshold may not be reached at stimulus offset. DIANA’s decision component then selects the candidate with the highest activation and expresses the ambiguity within this selection process in terms of additional reaction time.

DIANA defines the reaction time for a stimulus in these situations as the sum of the duration of the word (during which no decision could be made), a so called ‘choice reaction time’, and an execution time. The computation of the choice reaction time is based on Hick–Hyman law [64,65,66], which states that the more choices are available (expressed in terms of entropy), the longer it takes for a decision to be made. In [67], following a number of early and more recent behavioral studies [64,65,68,69,70], it is shown that the Hick–Hyman law has a neural underpinning in the cognitive control network (CCN) and the default mode network (DMN), which deal with the mental representation of uncertainty and the generation of behavioral responses [71,72] and which support adaptive behavioral control across a broad range of cognitive demands [73,74,75,76]. It appeared that the entropy of the decision problem increased the activity of the CCN that is involved in uncertainty processing and response generation, and decreased the activity of the DMN, which is only involved in uncertainty representation. In short, these studies provide a neurophysiological link between entropy in a choice to be made on the one hand, and associated response latencies on the other. From this point of view, entropy may well explain delays in reactions.

The entropy which forms DIANA’s basis for the computation of the choice RT takes the activation scores of all candidates (words, pseudo-words, parts of words) into account, after removal of nested variants with lower activations from the competitor list. More details about the entropy computation can be found in Appendix A.4.

## 5. Future Research Directions

DIANA is transparent about all processes and assumptions, both at the conceptual and computational level. Transparency, in combination with a process-oriented account, provides clarity about what exactly DIANA can explain and account for. Neurological arguments play a guiding role in DIANA’s design; both for the activation and the decision components, the conceptual choices are based on neurophysiological findings (such as the role of STRFs in the auditory cortex, and the neurological underpinning of the Hick–Hyman law). In this section, we will illustrate a number of future research directions for improving DIANA, in particular the structure and content of its lexicon, the computation of the top-down information, the aspect of learning, and several implementation choices.

### 5.1. Lexicon

As described in Section 3.2, the pronunciation of words in DIANA’s internal lexicon is defined in terms of phone sequences. Words sharing a phone subsequence in the lexicon share the articulatory model pertaining to that subsequence. This structure is adequate insofar as differences between words can be expressed at the phone level. It cannot model subtle acoustic differences between the phone sequences that words share. For instance, the present structure of the lexicon cannot capture effects due to prosodic lengthening which listeners may be sensitive to (e.g., [30]). Similarly, homophones, such as ‘time’ and ‘thyme’, have in DIANA’s present lexicon identical representations, and the present structure of the lexicon, therefore, cannot deal with durational differences among the members forming a homophone pair [77]. Note that DIANA can *detect* duration differences in the acoustic signal; duration modeling is performed via the transition and self-loop probabilities of the hidden Markov models. The question then arises of whether and to what extent to incorporate these ‘fine phonetic cues’ in the mental lexicon, or, in other words, how to make DIANA’s word recognition sensitive to fine phonetic details insofar as they are perceptually relevant [78]. One of the options is to completely disentangle the representations of the different lexical entries, such that ‘discolor’ and ‘discover’, ‘time’ and ‘thyme’, ‘ham’ and ‘hamster’, and so on, do not share any common spectro-temporal structure. Such an option raises the question of where the detailed pronunciation information to be incorporated in the lexicon has to come from. It requires the analyses of either speech corpora in which the prosodic and spectral differences can be inferred in a statistically and perceptually significant way, or an implementation of a solid theory about the morphological-acoustics interface.

Another shortcoming of DIANA’s present structure of the lexicon is that each word is considered a separate, independent entry. This implies that, for instance, morphological information about shared stems is missing and that DIANA cannot model the influence of family size on the speed with which a word is recognized (e.g., [79]). These types of effects could be accommodated in a model of the lexicon where words are interconnected on the basis of all kinds of similarities (morphological, phonological, pragmatic, syntactical), as proposed by Bybee [80]. One of the future research directions is therefore the enrichment of DIANA’s lexicon by designing a network in which words are linked in a weighted fashion on the basis of all these different similarities. This network will modulate both the set of word candidates considered during the search and their activations. Connecting words on the basis of formal similarities (e.g. phonological or morphological) is a relative easy step compared to connecting words on the basis of their semantics. The latter may require that, in DIANA’s lexicon, words are coupled with semantic (distributed) representations (see also [81]).

### 5.2. Generalizing to Other Languages

So far, DIANA has been tested with Dutch and English [2,3]. This raises the question of to what extent it can also perform well with typologically different languages. One challenge is presented by languages that are morphologically more complex than Dutch and English, such as Finnish. They form a challenge because the fact that the same stem may be incorporated in a very high number of words increases the necessity of a flexible way of incorporating morphological structure, moving away from the current ’localist’ approach in DIANA in which each word form is represented as a single entry in the lexicon. Testing DIANA on such languages is on our agenda.

Another challenge is formed by tone languages. In the current version, DIANA is insensitive to pitch and to tone. Tone languages will ask for an extension of the acoustic feature extraction with pitch-related vector components (e.g., pitch itself, its first time-derivative). This is feasible since this extension has been incorporated in several speech decoding systems, for example, Mandarin [82]. To what extent the decoding approach in DIANA is compatible with the lexical structure of tone languages is another topic to be investigated in more detail.

In its current implementation, DIANA is monolingual. In principle, DIANA can also simulate multilingual listeners. In a multilingual setting (see, e.g., [83]), the potential number of competitors is much larger than in a monolingual listener, as multiple lexicons are activated simultaneously. As a consequence, the competition will be more involving, especially if stimuli are presented without any pre-context indicating the language. How this could be accomplished in DIANA is a challenging topic of further research.

### 5.3. Top-Down Information

Word activations result in DIANA from a combination of bottom-up information and top-down information. In the present version of DIANA, the top-down information is provided via a conventional statistical language model (SLM, in the form of an N-gram [49]) that estimates the (scaled) log probability of each word given the directly few preceding words (or given its frequency of occurrence when the word is presented out of context). The value of *N* depends on the available type of text materials; in the case of a list of isolated words, N=1 (unigram). Previous work has shown that these types of models predict reasonably well the following word [48]. However, these models may be argued to be cognitively too simplistic, as these models only consider a few preceding words, ignore the meanings of the words, ignore the syntactic structure of the sentence, and so on.

We aim to enrich the present top-down information in several ways. First, we will expand the number of preceding words that are taken into account by replacing the simple statistical language model by, for example, LSTM-based neural network-based language models (e.g., [49,84]) which can capture longer span word prediction. Second, we aim to produce expectations about the likelihoods of the different parts of speech, extracted from tagged corpora (for example by a modern dependency grammar approach, e.g., [85]). Third, we aim to enrich the top-down information with the meanings of the preceding words, expressed, for instance, in word2vec [33]. In further steps, the likelihoods of words could even be modulated by visual information presented to DIANA, as is done in image–caption retrieval models, such as [86].

### 5.4. Is DIANA A Learning Model?

One may require from a model that it not only simulates adult listener’s processing, but also how this adult acquired the knowledge to do so (language acquisition) and how this adult can learn new words and pronunciations. Language acquisition is a process mediated by social interaction in a multi-modal context that enables infants and toddlers to infer associations between acoustic forms and meanings with as a side-effect a capability to break up stretches of speech into words, syllables and sounds. It has been shown that all representations currently used in DIANA could be acquired incrementally [87,88,89,90,91,92,93,94], see also [95]. This paves the way to advance DIANA in the direction of an ecologically defensible model of speech comprehension. The present implementation of DIANA, however, lacks the capability of automatically learning new words, or new, deviant pronunciations of words that are already in the lexicon. We consider the aspect of dynamic word learning as a very relevant way to proceed. Conceptually, this word acquisition process could be associated with the detection of a pseudo-word in the sense of an out-of-vocabulary word, in combination with the inclusion and consolidation of the new form into DIANA’s lexicon. How this could be achieved is a topic for further research.

The present version of DIANA needs specifications of the probabilistic relations between MFCC feature vectors and STRFs. The question may be raised of how these are ‘learned’ by DIANA. We derived these low-level parameters by some kind of iterative optimization procedure using a large transcribed speech corpus. Obviously, this iterative, corpus-based approach is not a realistic proxy for language acquisition. DIANA could learn the low-level parameters incrementally, but doing so would be time consuming, and it would most probably contribute little to the insights that can be gathered with the current implementation in adult word recognition.

### 5.5. Implementation

The activation component in DIANA used in previous experiments (e.g., [2,6]) relied on speech analysis and decoding algorithms in the HTK software package, which can also be applied for automatic speech recognition [96]. The present version of DIANA [7,52] uses algorithms from a different software package, the KALDI toolkit [97]. The most important advantage of KALDI over HTK is the availability of more flexible tools for handling lattices that contain the dynamically changing activations of word and pseudo-word candidates as the acoustic stimulus unfolds. Another advantage of KALDI is that it allows lexicons with a practically unlimited number of entries, whereas the lexicon size in the HTK-based implementation was limited to about 25,000 entries.

Although parts of DIANA are built upon speech decoding algorithms that can also be used for automatic speech recognition, DIANA cannot be regarded as a variant of automatic speech recognition. The way in which activations are computed as functions over time is different, the way in which short input signals may activate longer words is entirely different, and DIANA’s activation computations are more neurologically inspired by the use of STRFs. Fully neural-network inspired approaches (such as EARSHOT) may stimulate the development of a variant of DIANA in which not only the representations (e.g., STRFs) but also processes are neurally informed. Considerations, as put forward by [36,37,38] about restrictions on relations between the implementation on lower and higher Marr levels, will be guiding in this direction.

## 6. Conclusions

This article presented DIANA, a process-oriented computational model of human word recognition. It differs from many models in that its input is the same acoustic signal as enters the human ear and in that its output are the outputs that can be produced by human participants in psycholinguistic experiments, so that DIANA’s plausibility can be directly tested. More importantly, DIANA’s design accounts better for the recent findings in neurophysiological and psycholinguistic research than previous models. Most importantly, DIANA does not assume a pre-lexical layer in which hard decisions are made about abstract pre-lexical units before lexical access, but converts the acoustic signal into representations (i.e., spectro-temporal receptive fields) that are neurophysiologically attested. In addition, DIANA resolves ambiguity following the Hick–Hyman law.

These features also imply that DIANA is fundamentally different from ASR models, including those based on deep neural networks. As a consequence, with DIANA we have a cognitively more plausible model of word recognition, which makes it easier to test new hypotheses about the human word recognition process in a cognitively valid way.

DIANA is work in progress. We have published several short papers on older versions of the model, mostly focusing on aspects of its implementation. In the present article, we have focused on the conceptual choices we made for DIANA, which resulted in those implementations (which are described in more detail in Appendix A). In the near future, we hope to further extend DIANA such that it reflects even better everything that is known about the human word-recognition process. We trust that, also in its present version, among the recently proposed computational models, DIANA can play a seminal role for the advancement of process-based accounts of human word recognition.

## Figures and Tables

**Figure A1:**
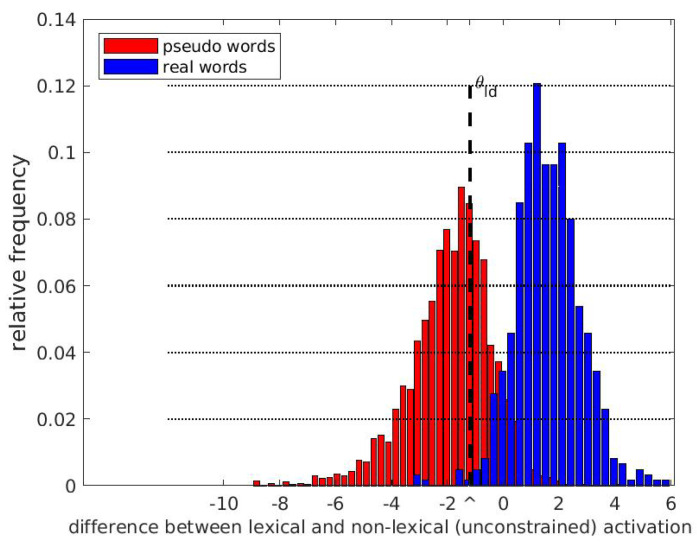
Histogram of the difference of lexical activation and non-lexical activation of 2780 existing words (blue) and 2761 pseudo-words (red) in BALDEY. The dashed vertical line indicates the lexical decision threshold (denoted θ with subscript ld, lexical decision); its optimal value depends on the task. In the figure, it is chosen such that the probability of words erroneously receiving a pseudo-word label would be minimal.

**Figure A2:**
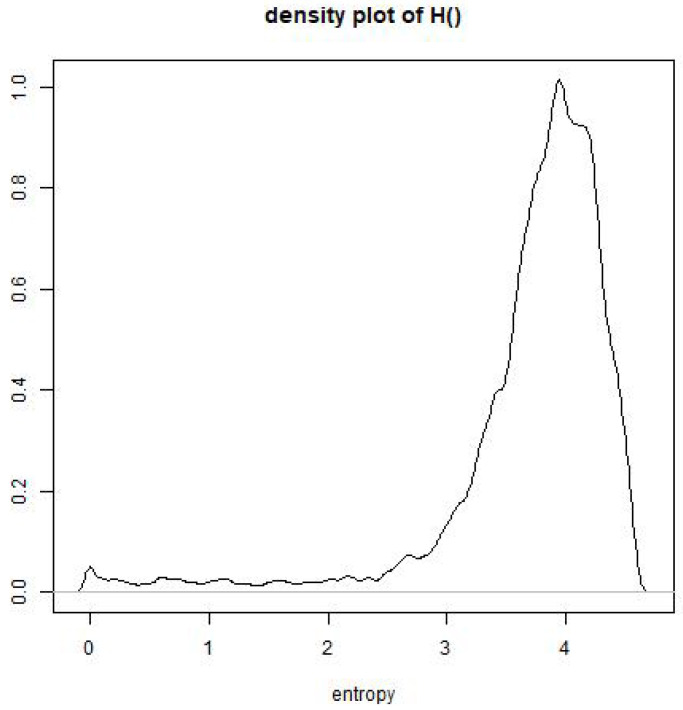
Density of the entropy H() over all 5541 BALDEY stimuli.

**Figure A3:**
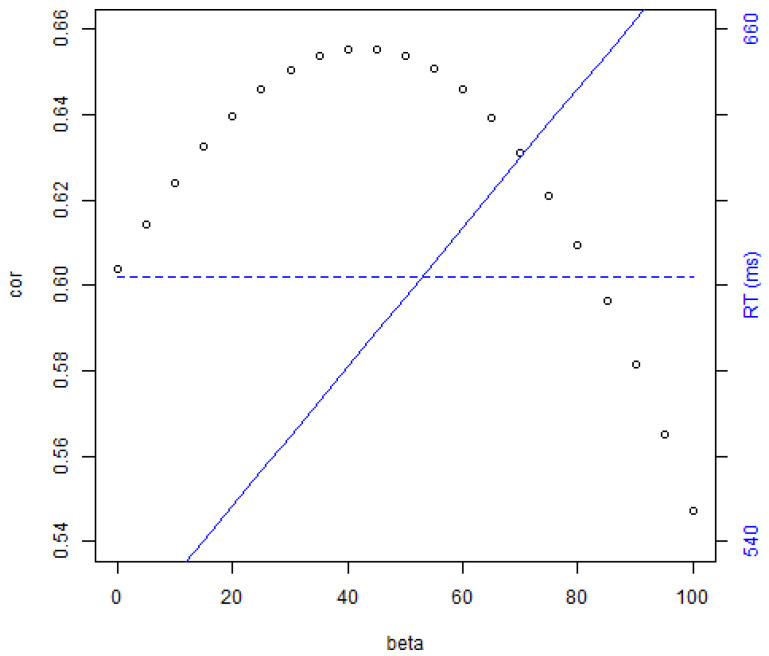
DIANA’s simulations for a word identification task. The dataset is a morphologically homogeneous subset of BALDEY. The black dotted curve shows the correlation between DIANA’s RTs and the average RT of participants as a function of beta (β), the coefficient of the entropy in the equation for DIANA’s RT. The solid blue line shows the average of DIANA’s RT as a function of β, while the dashed blue horizontal line indicates the participant’s mean RT. The right-hand side vertical axis pertains to the blue curves.

## Data Availability

The version of DIANA described in this paper uses several tools from the Kaldi toolkit. The STRFs used in the simulations and their relations to phonetic symbols were obtained using the Spoken Dutch Corpus. Contact the first author for the code.

## Abbreviations

    The following abbreviations are used in this manuscript:

ANN	artificial neural network
CCN	cognitive control network
DMN	default mode network
DNN	deep neural network
ECoG	electrocortocography
STRF	spectro-temporal receptive field
MFCC	mel-frequency cepstral coefficient
RT	reaction time
LM	language model
SLM	statistical language model
NDL	naive discriminative learner
LDL	linear discriminative learner

## Appendix A. Appendices

The four appendices below provide more detailed information about the computational approach underlying DIANA. In Appendix A.1, we discuss the statistical relation between MFCC feature vectors and spectro-temporal receptive fields. In Appendix A.2, we discuss the activation of words, pseudo-words and word cohorts. In Appendix A.3, we discuss the procedure for modeling lexical decisions. Finally, in Appendix A.4, we discuss various aspects of DIANA’s entropy.

### Appendix A.1. MFCC Feature Vectors and Spectro-Temporal Receptive Fields

In DIANA, the competition between words, parts of words and pseudo-word candidates is determined by the degree of match between the audio input and the internal representations of these candidates. The audio input is represented as a sequence of mel-frequency cepstral coefficient (MFCC) feature vectors [48]. Any candidate is, via its phone sequence representation and the corresponding hidden Markov model, represented as a sequence of so-called spectro-temporal receptive fields (STRFs) (see, e.g., [34]). Each Markov state is associated to an STRF. The match between the signal and the candidate is then defined by the statistical match between the MFCC sequence and the STRF-based Markov model.

Each individual MFCC feature vector is based on a speech segment of 25 ms wide, and is updated with a 10 ms time shift sliding through the speech signal. In this feature vector, both static and dynamic parts in the speech signal are accounted for [48]. For an utterance, this vector sequence forms a trajectory in the MFCC space, sampled 100 times a second. Obviously, new realisations of the same utterance (even when uttered by the same speaker) may lead to slightly deviant trajectories which may be acoustically different but count the same on a phonemic level. This implies that neighboring MFCC feature vectors along a trajectory probably belong to the same speech sound.

This suggests a statistical relation P(STRF|MFCC) between MFCCs and STRFs. This relation is established outside of DIANA via a conventional forced-alignment procedure between audio recordings and a parallel phone-level annotation. This alignment defines each STRF as a statistical distribution (cluster) in the MFCC space. STRFs pertaining to states belonging to one phone are similar and may have a substantial overlap in MFCC space, while STRFs related to different phones may be very dissimilar. Because STRFs partition the MFCC space in a statistical way, each trajectory in the MFCC space passes though a number of MFCC regions, thereby activating the hidden Markov states via P(STRF|MFCC) (see Appendix A.2).

The use of phones in the alignment is not essential for creating a useful set of STRFs, neither is the forced alignment procedure (Viterbi algorithm). Other clustering methods, e.g., an unsupervised clustering of MFCC vectors, may yield equally useful STRFs, but the current choice for phones makes it possible to interpret DIANA’s output (e.g., the outputs in Section 3.4) and the candidates during competition in phonetic terms.

### Appendix A.2. Activation of Words, Pseudo-Words and Word Cohorts

The activation of word candidates, parts of words and pseudo-words is based on a combination of bottom-up evidence from the speech signal and top-down prediction. For the computation of this activation, Bayes is the starting point (similar to [25,98]):

(A1) P(W|S)=P(S|W)P(W)/P(S)

in which the left-hand side is the probability sought. The speech signal and the word candidate(s) are denoted by *S* and *W*, respectively. *S* is represented as a sequence of MFCC feature vectors, and *W* is represented by a Markov model, each Markov node modeled by an STRF.

In the computations, DIANA follows the actual practice in which the denominator P(S) is ignored since it is independent of the word candidate *W* (see [48]). This step, turning probabilities into likelihoods, makes sense for psycholinguistic experiments; what counts is the activation of some word (sequence) $W_{0}$ relative to the activation of competing word (sequences) $W_{m},i=1,\cdots ,M$. This allows us to ignore the prior probability P(S) of the speech signal from the equations, since it is the same for all candidates. Moreover, all computations are performed in the logarithmic domain, that is, in terms of log-likelihoods.

During the unfolding of the signal *S*, DIANA must not only be able to deal with gated input signals, such as ’cathedr’, but also with partial word candidates and pseudo-words. To compute on-line activations for word cohorts (parts of words starting at the beginning) and pseudo-words *W* while the acoustic signal *S* unfolds, DIANA extends Equation (A1) to:

(A2) logP(W|S[0:t])∼logP(S[0:t]|W)+logP(W)

in which S[0:t] denotes the gated part of the signal *S* up to time *t*, and *W* may be a complete word or word part or pseudo-word. The ∼-sign is used because this equation is actually in terms of log likelihoods. Due to the assumption of independence of subsequent hidden Markov states, logP(S[0:t]|W) in the right-hand side of (A2) can be written as a sum over sequences of vectors (MFCC feature vectors) and corresponding states (STRFs):

(A3) $$\log P\left(S\left[0:t\right]|W\right)=\sum_{vector,state}\log P\left(vector|state\right)$$

in which P(vector|state) is provided by the forced alignment that was used to construct the STRFs (Appendix A.1). As a result, logP(W|S[0:t]) is an accumulation of feature vector-based contributions from 0 to *t*.

In order to enable DIANA to compare activation scores of hypotheses with different durations, we perform a duration normalization. This accounts for the fact that longer utterances yield more acoustic evidence, but what essentially counts is the amount of evidence per unit time. This has a parallel with human speech processing in which the time window within which a listener may revise an hypothesis cannot be not arbitrarily long. This normalisation is performed by dividing the log likelihood by the duration *t* of the speech signal S[0:t]. By doing so, we obtain a duration-normalized log likelihood log(P(W|S[0:t])/t. This normalized value is referred to as the activation of a word at time *t*.

### Appendix A.3. Lexical Activation Score and Lexically Unrestricted Activation Score

To simulate a participant’s lexicality judgments in a lexical decision experiment, DIANA adopts a strategy for making a word or pseudo-word decision. In a word identification task, words go into competition with other words. This situation is different from lexical decision, in which the competition is between words on the one hand and pseudo-words on the other. Since the criteria for word decisions may be different from the criteria for pseudo-word decisions [99], DIANA uses a strategy based on the comparison of two activation scores that are computed in parallel, a lexical score (based on all words in the lexicon) and a lexically unrestricted score (based on all phonotactically licensed phone sequences), for each gated signal S[0:t] for all *t*:

(A4) $$argmax_{any\;lexical\;form}\log P\left(form|S\left[0:t\right]\right)/t$$

the lexical score, and 

(A5) $$argmax_{any\;phonotactically\;licensed\;form}\log P\left(form|S\left[0:t\right]\right)/t$$

the lexically unrestricted score.

The results of the computation of the first, ’lexical’, activation are presented in e.g., Figure 4 and Figure 5. For the computation of the second activation, DIANA’s search space is dynamically enlarged to allow all phonotactically licensed word forms, such as ’cath’, ’cathedral’, ’cathedruke’, ’thedruke’, etc. DIANA can create such pseudo-words as word hypotheses on the fly, on the basis of a phone network in which phones are represented as nodes, such that only those phone combinations that are phonotactically licensed appear as possible paths through the network. With respect to the computation of activation scores, pseudo-words or word parts do not behave in a principally different way from real words.

Conceptually, both the lexical and lexically unconstrained activation scores make sense, albeit in different ways; the lexical activation is the key ingredient in the word-to-word competition in speech comprehension of known words, while the second activation is at stake when listeners are confronted with unknown words (e.g., new names) or pseudo-words. Recent studies using EEG analyses [17] show how listeners can take recourse to a phonological grammar to process unknown words. This is related to DIANA’s network-based strategy (described above) underlying the lexically unrestricted activation score.

If all top-down predictions are equal, the second score is always at least as good as the first, since the lexically constrained phone sequences form a subset of the phonotactically licensed phone sequences. This implies that the difference between these activations is an indication for the lexicality of the stimulus. DIANA’s use of a decision criterion based on the difference between two activations is commonly used in general models of human decision and RT distributions, e.g., the ballistic accumulation model (BAM) [54] and the linear approach to threshold with ergodic rate models (LATER, [55,56]). For several reasons, among others the effect of between-speaker pronunciation variation and the probabilistic relation between MFCCs and STRFs, it is not guaranteed that the activation score difference distinguishes lexical from non-lexical inputs in a fully reliable way. Inevitably, this will give rise to lexicality judgment errors.

Figure A1 (see also [1,2]) shows the distributions of the lexical activations for existing words (in blue) and the lexically unconstrained activations for pseudo-words (in red) of the 2780 existing and 2761 pseudo-words in BALDEY [63]. For each stimulus, the difference between the highest lexical and the highest non-lexical activation is displayed on the horizontal axis. The dashed vertical line represents the position of a criterion value θ (a model parameter) that can be used as a threshold to decide the lexical status of a stimulus. If the difference between lexical and non-lexical activation exceeds θ, the stimulus is classified as a real word, otherwise it is assumed to be a pseudo-word. The expected classification error will depend on the structure of the pseudo-word stimuli in a lexical decision experiment and the exact way they violate lexicality. For example, in stimulus sets in which pseudo-words only deviate from real words in one phone, the blue and red distributions might overlap to a large extent (due to the small acoustic difference between the pseudo-word and its closest real word). In the case of clear acoustic differences between pseudo-words and real words, the blue and red distribution would hardly overlap.

### Appendix A.4. Computation of Entropy

In DIANA, entropy, a measure of the ‘degree of disorder’ in a physical system or of the ‘complexity of a decision process’, is assumed to be a contributing factor to the long latencies manifest in many psycho-linguistic tasks. During the word search, the entropy is computed from the word probabilities, which are computed from the scaled log likelihoods logP(W|S[0:t]) in Equation (A2). This is performed for each *t*, such that entropy values are available as a function of time. To compute the probabilities from the scaled log likelihoods, DIANA used a procedure that is similar to the probability normalization step often applied in speech decoding research (A6). In this procedure, the scaled log likelihoods are first transformed into unscaled log likelihoods, by applying a multiplication with a constant *c* (which is to be estimated from data). Next, the ‘softmax’ Luce rule is applied to convert the log likelihoods to probabilities, via normalisation to make the sum equal to 1. In total:

(A6) $$p_{W,t}=\frac{\exp(c\cdot \log P(W|S[0:t]))}{\sum_{W}\exp\left(c\cdot \log P\left(W|S\left[0:t\right]\right)\right)},$$

for each time point *t*. The sum in the denominator runs over all hypotheses viable at time *t*. The value of *c* is estimated to be approximately −0.05, by using the word-confidence estimation approach described in [100]. This value is an approximation; a more precise value can be obtained if the list of candidates is made more precise. The value of *c* is not critical for the evaluation of the entropy. Similar methods are also applied to compute word confidence measures [101]. In DIANA the entropy was computed after removal of all ‘nested’ hypotheses from the list of hypotheses (see Section 4). Although the conceptual aspects underlying this procedure are transparent, the required computations are often quite technical in nature.

#### Appendix A.4.1. Entropy as a Contributing Factor of Reaction Time

Reaction times in a behavioral experiment are a good example of outcomes of a complex cognitive process in which many factors play a role at various time scales (e.g., [3,7,12,102,103,104]. One of the factors that is likely to play a role in the explanation of reaction time is the complexity of the problem that a participant must solve while making a decision. In lexical decision tasks, the RT distributions are typically very skewed with a long tail towards long RTs [63,105]. For example, in the large-scale Dutch lexical decision database BALDEY [63], which contains over 110,000 lexical decisions and reaction times, only a very small subset of the stimuli receive a valid reaction before the end of a stimulus, about 50% of all RTs (when measured from stimulus offset) exceed 510 ms, and 10% exceed 1560 ms. Moreover, Ref. [105] reports similarly substantial RTs. These long tails indicate that participants may need a substantial amount of time to make a decision, since the time that elapses between stimulus offset and an overt response (e.g., a button press) largely exceeds the neural traveling time required to effectuate an overt response (which is 200 ms at most).

The long tail in RT distributions form a challenge for modeling. In regression models of RT, the dependent variable is often transformed using log(RT) or the inverse (1/RT), which makes the distribution of the transformed RTs more Gaussian-like. In BAM and similar models [54,56], the skewness is dealt with by putting constraints on the distribution of the drift rate. DIANA, as a process-oriented model, must be able to explain reaction times far beyond the stimulus offset on the basis of activations that are computed before stimulus offset. This is performed by relating the additional reaction time with the complexity of the choice. DIANA takes the Hick–Hyman law as a starting point [64,65,66]. In the case of *N* options with equal probability p=1/N, the time necessary to choose one option is linear in the log-transformed number of items:

(A7) $$\Delta T=A+B\cdot \log\left(N\right)=A+B\cdot \sum_{all\;N\;items}-p\log\left(p\right)$$

with *A* and *B* constants that depend on the details of the experiment. In DIANA, this situation is translated into the choice a listener has to make among *N* hypotheses, each with different probabilities (*p*, from Equation (A6)). To that end, the right-hand term in Equation (A7) is generalized to the entropy $\sum_{i=1}^{N}-p_{i}\log\left(p_{i}\right)=H\left(p_{1},...,p_{N}\right)$. DIANA’s *choice RT*, the contribution to the overall RT due to entropy, is then modeled as

(A8) $$choice\;RT=\beta \cdot H(p_{1},\ldots ,p_{N})$$

in which the factor β>0 is one of the meta-parameters in DIANA, translating entropy into additional reaction time, and $p_{i}$ are the probabilities of the hypotheses as derived from Equation (A6). A larger degree of ambiguity (e.g., more close competitors, very similar words in the competition, pseudo-words close to real words) leads to larger entropy and so will increase the reaction time. In the case that there is no ambiguity, *H* equals zero and so the choice RT vanishes.

When entropy is taken into account, one option to express DIANA’s RT predictions beyond stimulus offset reads as follows:

(A9) $$\begin{array}{ccc} DIANA\;RTonset & = & stimulus\;duration+\beta \cdot H()+\\ & & f(morpho-syntactic\;factors)+\\ & & execution\;time \end{array}$$

in which DIANA’s meta-parameter β translates entropy H() to additional choice RT (via $choice\;RT=\beta \cdot H(p_{1},\ldots ,p_{N})$). Here, *f* denotes a zero-mean (as yet unspecified) function that modulates the reaction time on the basis of morpho-syntactic factors of the stimulus. In the following subsection we show that this option is supported by regression modeling, by simulating data on a morphologically homogeneous subset of BALDEY, such that the variation in *f* is limited.

An even more challenging option for integrating entropy would be in line with what has been discussed in the Discussion section concerning the extension of DIANA’s lexicon with morphologically and semantically relevant information:

(A10) $$\begin{array}{ccc} DIANA\;RTonset & = & stimulus\;duration+\\ & & \beta \cdot H(morpho-syntactic-semantic\;factors)+\\ & & execution\;time \end{array}$$

in which first morpho-syntactic properties, such as stem sharing, family size effects, and semantic relations, modulate the hypotheses’ probabilities, on the basis of which a new entropy H() is computed.

Figure A2 illustrates the distribution of the entropy (a density plot) as computed in DIANA’s decision component for all 5541 auditory stimuli used in BALDEY [63]. The distribution shows a tail towards 0. This tail is due to relatively long stimuli, some of which have no—or at best very few—competitors left at their offset. The mismatch between the highly asymmetric distribution of entropy values (long tail towards the lower values) and the fairly symmetric distribution of log-transformed RTs shows that entropy on its own may not be a very powerful predictor of RTs, but nevertheless may serve as a significant predictor of RTs in regression models. An example is shown in the next subsection.

#### Appendix A.4.2. Entropy as a Predictor of the RTs in BALDEY

To illustrate the impact of DIANA’s entropy computed at offset of the acoustic stimuli in predicting RT values, we present one linear mixed-effects [106] -based regression analysis using the data in BALDEY [63]. In DIANA, it is assumed, via Hick’s law, that a larger entropy leads to longer reaction times when all other factors are assumed equal, while taking into account the challenges connected to this interpretation as voiced in [66]. If true, this should correspond to the entropy having a positive regression coefficient in models of RT.

Since RT distributions typically comprise some extremely fast, and a long tail of very slow, reactions that arguably are not representative of the ‘normal’ cognitive processes, we ignored all trials with an RT measured from stimulus onset (RTonset) in the lowest percentile (pertaining to an RT threshold of 550 ms) and all RTs beyond μ+2σ. This step removed approximately six percent of the BALDEY trials, respectively. In the analysis, we further limited the trials to those BALDEY stimuli that were correctly responded to. The reaction times were log-transformed and served as the dependent variable.

We also added the predictor ‘wordiness’, defined as the ratio of the activation of the best lexical candidate and the activation of the winning candidate (word or pseudo-word). Furthermore, we added conventional predictors (see also [63]), such as logwdur (log-transformed word duration), logFreq, session, trial, wordclass, and compoundtype. The predictor wordclass is a factor with three levels (adjective, noun, and verb, with, in BALDEY 22,154, 48,861 and 32,917 tokens, respectively). ‘Adjectives’ are on the intercept. Compoundtype distinguishes four types of compounds (simple, adj+noun, noun+adj, and noun+noun). Here, ‘simple’ is on the intercept. Since the correlation between logwdur and entropy was r=−0.51, entropy was residualized over log word duration, with entrlogwdur as a new predictor. Finally, we added two predictors prevBVis and maRT [104,107] as control predictors. These predictors model the local trends that exist in human RT sequences that are independent of the stimulus itself but have a mid-term range of about 10–20 stimuli due to, e.g., learning effects, fatigue and fluctuating attention (for details about prevBVis and maRT, see, e.g., [104]).

Model search was by backward elimination starting from a regression model with all predictors and plausible interactions in the fixed structure and control predictors as random slopes without interactions. The final lmer model reported here has been derived according to the guidelines in [102,108,109]. Insignificant interactions and main predictors are left out of the final model. Random slopes were only included insofar as the resulting models converged and the AIC value was improved. Table A1 presents the results of the final lmer model for RTonset. The AIC of this model (m) equals −753.2811.

$$\begin{array}{cc} m\;= & \;\mathrm{lmer}(\mathrm{logRT}\;\sim \;\mathrm{logwdur}\;*\;\mathrm{logFreq}\;+\;\mathrm{entrlowdur}\;+\;\mathrm{wordiness}\;+\\ & \mathrm{session}\;+\;\mathrm{trial}\;+\;\mathrm{wordclass}\;+\;\mathrm{maRT}\;+\;\mathrm{prevBVis}+\;\mathrm{compound}\_\mathrm{type}\;+\;(1|\mathrm{word})+\\ & (1\;|\mathrm{subject})\;+\;(0+\mathrm{maRT}\;|\mathrm{subject}),\\ & \mathrm{data}\;=\;\mathrm{data}4[\mathrm{data}4\$\mathrm{response}\;==\;“\mathrm{correct}”,]) \end{array}$$

**Table A1:** Output of lmer model modeling RTonset, including the DIANA-based predictors entrlogwdur, which is the entropy residualized over log word duration, and wordiness. For details see the text.

RT_onset_			
	Estimate	Std. Error	*t* Value
(Intercept)	6.742 × $10^{-1}$	3.265 × $10^{-1}$	2.065
logwdur	3.306 × $10^{-1}$	6.513 × $10^{-3}$	50.760
logFreq	7.011 × $10^{-2}$	1.135 × $10^{-2}$	6.178
entrlogwdur	2.354 × $10^{-2}$	2.039 × $10^{-3}$	11.548
wordiness	5.559 × $10^{-2}$	9.084 × $10^{-3}$	6.119
session	2.564 × $10^{-3}$	2.919 × $10^{-4}$	8.783
trial	2.672 × $10^{-5}$	4.883 × $10^{-6}$	5.472
wordclass(nom)	6.933 × $10^{-3}$	2.965 × $10^{-3}$	2.338
wordclass(verb)	3.106 × $10^{-2}$	2.951 × $10^{-3}$	10.526
maRT	6.000 × $10^{-1}$	4.299 × $10^{-2}$	13.958
prevBVis	2.651 × $10^{-3}$	2.883e × $10^{-4}$	9.196
compoundtype(A+N)	−3.023 ×$10^{-2}$	8.855 × $10^{-3}$	−3.414
compoundtype(N+A)	−7.375 ×$10^{-3}$	1.018 × $10^{-2}$	−0.724
compoundtype(N+N)	4.197 × $10^{-3}$	4.236 × $10^{-3}$	0.991
logwdur:logFreq	−1.277 ×$10^{-2}$	1.785 × $10^{-3}$	−7.153

The final model for RTonset was significantly better (in terms of AIC) than alternative models, including models in which entrlogwdur or wordiness were left out. Table A1 provides regression coefficients and *t*-values of this model; under the assumption of the validity of the *t*-distribution, all values of |t|>1.96 are considered to indicate significance at the level p<0.05. In this model the two-way interaction between logwdur and log frequency is kept, and maRT serves as a random slope under subject without correlation with the intercept. As expected, (log) word duration (logwdur) is one of the most significant predictors of reaction time measured from stimulus onset.

Interestingly, the Table shows that both DIANA-related predictors (entrlogwdur and wordiness) are significant, with positive βs. A larger entropy (measured at stimulus offset) thus leads to a larger reaction time, all other factors being equal. The positive β for wordiness shows that the more probability mass is attributed to word hypotheses, the slower the decision. This seems counter-intuitive, since one would expect that the larger the probability mass attributed to one hypothesis, the faster the decision should be. However, wordiness is the score of the top-ranking hypothesis, irrespective of whether that is an existing word or a pseudo-word. It appears that the number of cohorts formed by sequences of transcription symbols resulting from trying the ‘transcribe’ pseudo-word stimuli is much smaller than the number of cohorts that make up the words in a large lexicon.

The other coefficients can be explained on the basis of earlier findings (e.g., [63]). Higher word frequency leads to smaller RTs, but modulated by an interaction with word duration. In general, later sessions and trials lead to slower RTs. Compared to adjectives, nouns and verbs yield longer RTs. Noun-noun compounds produce the slowest RTs among compounds. The ‘local trend’-related control predictors maRT and prevBVis are highly significant with β>0, again showing the usual substantial local speed effect [104].

Finally, Figure A3 shows the predictions of DIANA when simulating a word identification task. The dataset was chosen to be a morphologically homogeneous subset of BALDEY (real words, morphologically simple, bi-syllabic), such that morphological factors are factored out as much as possible (see also [1,2,3]).

The figure shows the performance of DIANA via Equation (A9) in terms of the correlation between DIANA’s RT sequences and the average RTs from participants in BALDEY (the dotted black line) and the average RT (solid blue line), both as a function of the coefficient β in Equation (A9). The black dotted line shows that the highest correlation between DIANA and the participants is obtained for a value of β>0 (ρ_max_ = 0.651, 95% confidence interval [0.641–0.663]), i.e., significantly higher than the prediction without entropy (β=0).

The dashed blue horizontal line indicates the participant’s mean RT. The right-hand side vertical axis pertains to the blue curves. The figure suggests that there is no single value for β for which both the correlation is optimal (highest point in black curve) and the average RT prediction is correct (crossing of solid and dashed blue lines). This strongly indicates that the operational definition of entropy, as currently used, can be refined, by, e.g., taking into account more complex (morphologically oriented) word–word relation during DIANA’s word competition stage, in line with the discussion of Equations (A9) and (A10).
